# Headache and Its Materia Medica

**Published:** 1889-02

**Authors:** 


### BOOK NOTICES AND REVIEWS.

### HEADACHE AND ITS MATERIA MEDICA. By B. F. Underwood, M. D. Pages 212. New York: A. L. Chatterton & Co. 1889.

As "headache" is met with so frequently and is often so difficult to relieve, we may well welcome any work which will aid us in curing this troublesome complaint. A headache promptly relieved will, perhaps, give a physician more reputation than the cure of a dangerous disease like typhoid or scarlet fever. It seems to be easier comprehended and more appreciated!

Dr. Underwood notices, first, the causes of headache as nervous, catarrhal, rheumatic, etc.; next, he gives indications for remedies. These are in the main well given; but it would have been better to have made a clear, succinct statement of the therapeutics of each drug, as stated in the materia medica, rather than give what physicians recommend.

After the therapeutics comes a brief repertory of head symptoms. As it is well to look at our therapeutics from all sides, it is probable that Dr. Underwood's monograph will be of assistance to some. It must not be forgotten, in prescribing for headaches, that we should prescribe for the whole patient, not for the head only; don't behead your patient!

